# The ‘Pokemon’ (*ZBTB7*) Gene: No Evidence of Association with Sporadic Breast Cancer

**DOI:** 10.4137/cmo.s569

**Published:** 2008-04-28

**Authors:** Antonio Salas, Ana Vega, Roger L. Milne, Manuel García-Magariños, Álvaro Ruibal, Javier Benítez, Ángel Carracedo

**Affiliations:** 1 Unidade de Xenética, Instituto de Medicina Legal, Facultad de Medicina, Universidad de Santiago de Compostela, 15782, Galicia, Spain; 2 Grupo de Medicina Xenómica, CIBERER, Universidad de Santiago de Compostela, Galicia, Spain; 3 Fundación Pública Galega de Medicina Xenómica (FPGMX)-Consellería de Sanidad Santiago de Compostela, 15706, Galicia, Spain; 4 Servicio de Medicina Nuclear, Hospital Clínico Universitario, Santiago; 5 Centro Nacional de Genotipado (CeGen), Centro Nacional de Investigaciones Oncológicas (CNIO), Madrid, Spain; 6 Programa de Genética del Cáncer Humano, CIBERER, Centro Nacional de Investigaciones Oncológicas (CNIO), Madrid, Spain

**Keywords:** breast cancer, *ZBTB7*, POKEMON, association study, case-control

## Abstract

It has been proposed that the excess of familiar risk associated with breast cancer could be explained by the cumulative effect of multiple weakly predisposing alleles. The transcriptional repressor FBI1, also known as Pokemon, has recently been identified as a critical factor in oncogenesis. This protein is encoded by the *ZBTB7* gene. Here we aimed to determine whether polymorphisms in *ZBTB7* are associated with breast cancer risk in a sample of cases and controls collected in hospitals from North and Central Spanish patients. We genotyped 15 SNPs in *ZBTB7*, including the flanking regions, with an average coverage of 1 SNP/2.4 Kb, in 360 sporadic breast cancer cases and 402 controls. Comparison of allele, genotype and haplotype frequencies between cases and controls did not reveal associations using Pearson’s chi-square test and a permutation procedure to correct for multiple test. In this, the first study of the *ZBTB7* gene in relation to, sporadic breast cancer, we found no evidence of an association.

## Introduction

It has been suggested that breast cancer, together with prostate and colorectal, are the cancers with the highest heritable components. A substantial proportion of familiar breast cancer (~25%) is explained by mutations in the *BRCA1* and *BRCA2* genes [1,2]. By contrast, the excess of familial risk associated with sporadic breast cancer (as well as the unexplained genetic risk in familial breast cancer) may be better explained by the effect of multiple weakly predisposing alleles [3,4]. The identification of common alleles conferring modest susceptibility to cancer (as opposed to the known high penetrance *BRCA1*/2 genes) is a field of growing interest, especially with the development of new genotyping techniques and SNP database facilities [5].

Hence, there is much interest in the search for low penetrance gene/variants for breast cancer, which could exist with relatively high prevalence in the general population. Many polymorphisms have been proposed as candidates for susceptibility to sporadic breast cancer but reported positive associations have rarely been replicated in independent studies [6–9].

Recently, Maeda et al. [10] identified the transcriptional repressor FBI1, namely Pokemon (POK erythroid myeloid ontogenic factor), as a critical factor in oncogenesis. This protein is encoded by the *ZBTB7* gene (“zing finger and BTB domain containing 7”; Gene ID: 51341). Mouse embyronic fibroblasts lacking *ZBTB7* are completely refractory to oncogene-mediated cellular transformation. Conversely, FBI1 over-expression led to overt oncogenic transformation both *in vitro* and *in vivo* in transgenic mice. FBI1 can specifically repress the transcription of the tumor suppressor gene ARF (600160). Maeda et al. [10] found that FBI1 is aberrantly over-expressed in human cancers, and its expression levels predict biologic behaviour and clinical outcome. On the other hand, tissue microarray (TMA) analysis in breast carcinomas has revealed high levels of Pokemon expression in a subset of these tumours. In addition, the genomic region where the *ZBTB7* gene resides (19p13.3) is a hotspot for chromosomal translocations (The Cancer Genome Anatomy Project; http://cgap.nci.nih.gov/). *ZBTB7* is therefore a good candidate low penetrance breast cancer susceptibility gene.

Here we aimed to study the potential implications of common *ZBTB7* variants in sporadic breast cancer in a sample of cases and controls from Spain. To do this, we selected a set of 19 SNPs covering the whole extension of *ZBTB7* and flanking regions at high density.

## Material and Methods

### Study subjects and DNA extraction

Cases were 360 Spanish women with breast cancer and mean age at diagnosis of 59 years (range 25 to 85 years), recruited between 2000 and 2004 (48% of cases were recruited within one year of their diagnosis and 79% within five years). All cases were collected from a consecutive series recruited via three public Spanish hospitals: *Hospital La Paz* (20%), *Fundación Jiménez Díaz* (50%) and *Hospital Monte Naranco* (30%). Our samples contain prevalently invasive cases of breast cancer, 96%; while only 4% of *in situ breast cancer*. Controls were 402 Spanish women free of breast cancer at ages ranging from 24 to 85 years (mean = 53 years) and recruited between 2000 and 2005, *via* the Menopause Research Centre at the *Instituto Palacios* (50%), the *Colegio de Abogados* (31%) and the Centro Nacional de Transfusiones (19%), all in Madrid. While data was not available to calculate response rates, our experience is that response rates are very high for cases (~90%).

Genomic DNA was isolated from peripheral blood lymphocytes using automatic DNA extraction (Magnapure, Roche) according to the manufacturer’s recommended protocols. DNA was quantified using picogreen and diluted to a final concentration of 50 ng/ul for genotyping. Informed consent was obtained from all participants and the study was approved by the institutional review boards of *Hospital Clínico Universitario* (Santiago de Compostela, Galicia, Spain) and *Hospital La Paz*, Madrid.

### SNP selection

SNPs were selected from different sources: the International HapMap Project (The International HapMap Consortium, 2003; 2004; http://www.hapmap.org/), Ensembl (Birney et al. 2004; http://www.ensemble.org/), the Sequenom RealSNP database (https://www.realsnp.com/default.asp), and PupaSNP (Conde et al. 2004; http://www.pupasnp.org/). All 22 SNPs described at the time of selection were included, which yielded an average coverage of 1 SNP/1.7 Kb. These SNPs cover the upstream and downstream flanking regions (10000 bp) and the introns of *ZBTB7*, and include only one coding non-synonymous SNPs (Table 1).

### SNP genotyping

Genotyping was performed using the MassARRAY SNP genotyping system (Sequenom Inc., San Diego, CA) located at the *Universidad de Santiago de Compostela* node of the Spanish National Genotyping Center (Centro Nacional de Genotipado; http://www.cegen.org), following the manufacturer’s instructions. This typing assay uses the extension of a single primer that binds to the sequence flanking the mutation site. Base-specific primer extension products are created 1–4 bases long depending on the substitution present. The different primer extension products are then differentiated by mass. Multiple sites can be typed simultaneously by multiplexing the extension reaction. Detection uses matrix-assisted laser desorption ionization time-of-flight (MALDI-TOF) mass spectrometry with samples automatically genotyped from each mass spectrum produced. The assays were designed using Spectro DESIGNER software. Case and control samples were genotyped using 384-well plates and automated protocols. The allele-calling of all possible SNPs in each DNA sample was performed automatically using SpectroTYPER–RT software. Positive and negative controls were incorporated in each genotyping plate in order to assess genotyping quality. We estimate a genotyping error rate below 0.001%.

### Statistical analysis

We tested for differences in allele frequencies between cases and controls using Pearson’s Chi-squared test (the best model is provided in Table 2). We adjusted for age in categories <45, 44–49, 50–54, 55–59, and ≥60 *via* logistic regression using Stata v8. Disequilibrium coefficients (D′) for adjacent SNPs were calculated using Haploview v3.11 [11]. We used Gold software [12] to graphically summarized patterns of linkage disequilibrium in *ZBTB7* because it is well suited to the analysis of dense genetic maps. Assuming a minimum allele frequency (MAF) of 3% (the average MAF of our SNP set) and a genetic effect of 2, the *a priori* power to detect association under a dominant model is above 70%.

Haploview v3.32 (http://www.broad.mit.edu/mpg/haploview) was used for estimating the genotyping coverage of the selected SNPs (see below) and haplotype block structure.

The Cocaphased program of the Unphased software package [13] was used to check for single SNP and haplotype associations. We tested all two-, three-, four-, and five-SNP haplotypes for association in a sliding window across the gene. The option ‘drop rare haplotypes’ was used in order to restrict the analysis to the haplotypes with a frequency >1%. We followed the permutation test procedure implemented in Unphased which provides *P*-values corrected for the multiple haplotypes tested. The EM algorithm was used to impute missing data.

Evaluation of stratification was carried out based on the genotyping of 28 neutral SNPs, as previously described in a separate study that targeted a different set of low penetrance breast cancer genes in overlapping samples [14].

## Results and Discussion

Three out of the 22 SNPs selected failed genotyping. Four out of the 19 remaining SNPs (namely, rs10405522, rs895330, rs350840, and rs350832) were successfully genotyped in less than 75% of the samples and were therefore excluded from association analyses. The average call rate for these 15 SNPs was 95% (see also preliminary results in [15–17]) and none gave evidence of deviation from Hardy Weinberg equilibrium. Table 1 summarizes their location and allele frequencies.

**Figure 1:**
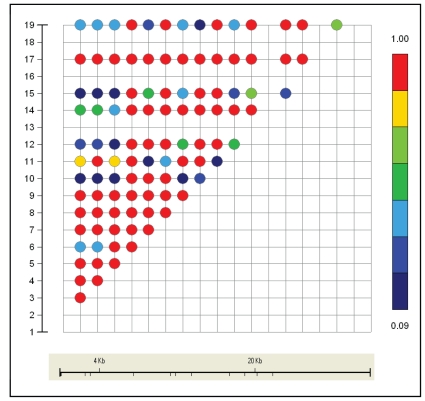
D′ pairwise linkage disequilibrium values of *ZBTB7* markers in control individuals.

**Figure 2:**
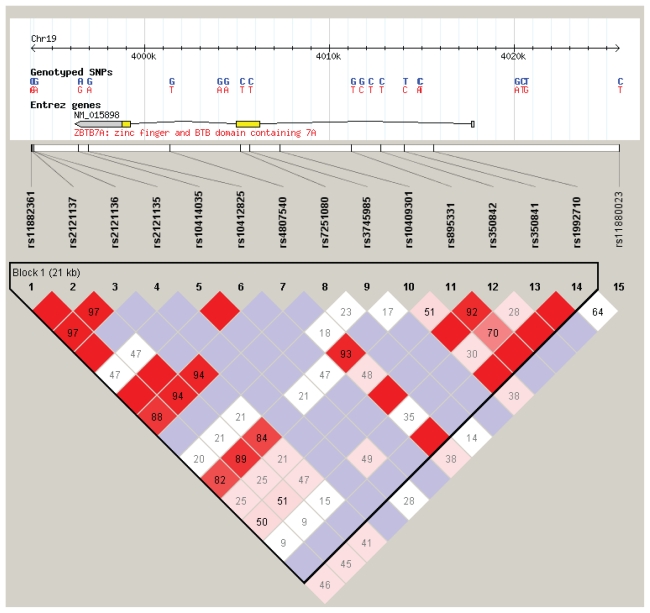
Haplotype block structure in our control individuals and HapMap information for the CEPH dataset (top).

We computed D′ values between all 19 markers, and detected moderate levels of LD (Fig. 1). However, under the ‘four gamete rule’ model (see Haploview for more information) we identified a haplotype blocks nearly covering the entire extension of the gene (Fig. 2). This characterization of LD along the *ZTBT7* region could be useful for future association study designs in cancer.

In order to measure the percentage of variability captured by the our selected SNPs, we first collected the HapMap data from the CEPH subset (http://www.hapmap.org) and the same chromosome range explored in the present study (chromosome 19: positions 3990056–4025697). Then, we estimated the number of SNPs un-captured in the CEPH-HapMap using our SNP selection under an r^2^ threshold of 0.8 and a model of ‘aggressive tagging’. Only one SNP in the HapMap dataset would remain untagged by our selected SNPs, indicating that our set of SNPs covers well the whole gene region under analysis.

No statistically significant differences between cases and controls were observed for individual SNPs based on comparisons of allele frequencies (see Table 2 for the best fitting models) whether or not age was adjusted for. Four- and three-SNPs haplotypes carrying markers rs350842 and rs350841 had associated *P*-values below 0.05 but were not significant after correction for multiple testing. Note also that these adjusted *P*-values overestimates the real value since the software employed (cocaphase) does not correct for the multiple hypothesis tested running different sliding windows.

To our knowledge, this is the first time that *ZBTB7* has been evaluated as a candidate sporadic breast cancer susceptibility gene. We have not found evidence of an association for *ZBTB7* SNPs nor haplotypes with breast cancer risk. It should be mentioned that most of the *ZBTB7* variants studied are rare in our sample. We are aware that the main drawback in detecting positive associations of rare variants (or haplotypes) is the need for large sample sizes. Therefore, the present result needs further validation in future studies of independent case-control series before a role for *ZBTB7* in breast cancer can be completely ruled out.

## Figures and Tables

**Table 1:** *ZBTB7* SNPs successfully genotyped.

ID	SNP	Alleles	MA	MAF (%)	Chr. 19 location in Ensembl v.31	aa change	Position (bp)	Intermarker Distance (bp)	Distance from v01 (bp)
–	rs10405522	C/G	G	2.4	3′-downstream	–	3990056	–	–
v1	rs11882361	A/G	G	2.9	3′-downstream	–	3993858	3802	3802
v2	rs2121137	C/T	T	2.6	3′-downstream	–	3993923	65	3867
v3	rs2121136	C/T	T	2.6	3′-downstream	–	3994024	101	3968
v4	rs2121135	A/G	A	0.2	3′-downstream	–	3994038	14	3982
v5	rs10414035	A/G	G	0.3	3′-downstream	–	3996434	2396	6378
v6	rs10412825	A/G	A	0.1	3′-downstream	–	3996962	528	6906
v7	rs4807540	C/T	T	4.0	intronic	–	4001399	4437	11343
v8	rs7251080	C/T	T	0.8	coding	341syn	4005208	3809	15152
v9	rs3745985	C/T	T	0.3	coding	A177T	4005702	494	15646
v10	rs10409301	C/T	T	4.7	intronic	–	4007346	1644	17290
v11	rs895331	G/T	G	2.1	intronic	–	4011222	3876	21166
–	rs895330	C/G	C	22.7	intronic	–	4011707	485	21651
v12	rs350842	C/T	C	1.1	intronic	–	4012781	1074	22725
v13	rs350841	A/G	A	0.9	intronic	–	4014067	1286	24011
–	rs350840	C/G	C	1.9	intronic	–	4015418	1351	25362
v14	rs1992710	C/G	C	0.2	intronic	–	4015672	254	25616
–	rs350832	C/T	C	22.9	5′-upstream	–	4020426	4754	30370
v15	rs11880023	C/T	T	21.1	5′-upstream	–	4025697	5271	35641

**Abbreviations:** MA: minor allele; MAF: control minor allele frequency; aa: aminoacid.

**Table 2:** OR and *P*-value for the best fitting model.

SNP	Rare Allele	Best Model	OR^1^	95% CI	Un-adjusted *P*-value
rs11882361	G	Dom.^2^	1.30	0.70–2.42	0.4
rs2121137	A	Dom.^2^	1.32	0.69–2.53	0.4
rs2121136	T	Dom.^2^	1.36	0.69–2.68	0.4
rs2121135	T	Dom.^2^	0.54	0.05–5.96	0.6
rs10414035	G	Dom.^2^	2.18	0.20–24.1	0.5
rs10412825	A	Dom.^2^	1.11	0.07–17.7	0.9
rs4807540	T	Dom.^2^	0.95	0.54–1.70	0.9
rs7251080	T	Dom.^2^	1.91	0.51–7.18	0.3
rs3745985	T	Dom.^2^	1.08	0.15–7.71	0.9
rs10409301	T	Rec.	0.27	0.06–1.29	0.1
rs895331	C	Dom.^2^	0.99	0.48–2.06	0.9
rs350842	C	Dom.^2^	0.70	0.24–2.08	0.5
rs350841	A	Dom.	0.78	0.24–2.47	0.7
rs1992710	C	Dom.^2^	0.52	0.05–5.81	0.6
rs11880023	T	Rec.	0.69	0.32–1.48	0.3

1 Using common-allele homozygotes as reference. The *P*-value refers to a Pearson’s Chi-squared test.

2 Only model that could be fit due to zero counts for rare homozygotes.
